# “We’re All in the Same Boat” – The Experience of People With Mental Health Conditions and Non-clinical Community Members in Integrated Arts-Based Groups

**DOI:** 10.3389/fpsyg.2021.661831

**Published:** 2021-03-17

**Authors:** Aya Nitzan, Hod Orkibi

**Affiliations:** Faculty of Social Welfare and Health Sciences, University of Haifa, Haifa, Israel

**Keywords:** community rehabilitation, arts groups, theater and drama, creative writing, music, mental health, stigma, recovery

## Abstract

In recent decades there has been a significant increase in community rehabilitation programs for people with mental health conditions. One such nationwide programs is *Amitim* in Israel whose mission is to foster the psychosocial rehabilitation of people with mental health conditions in the community. *Amitim*’s flagship program consists of arts-based groups that integrate participants with mental health conditions and non-clinical community members. To better understand the experiences of participants in these arts-based groups, five focus groups were conducted with participants from 15 integrated arts-based groups. In total, 17 people with mental health conditions and 21 non-clinical community members were interviewed for this qualitative study. Three main themes emerged from the thematic analysis: creation and expression through the arts promote well-being, self-disclosure in a safe space encourages a sense of belonging, and “we are all in the same boat.” The participants underscored the role of creation and expression through the arts in facilitating emotional expression, self-discovery, interpersonal communication, and spiritual elevation. The findings suggest that the facilitators should instill a sense of equality by enabling intergroup acquaintances without labeling participants’ mental health status. Integrated arts-based groups should be accompanied by a mental health professional who can contain and work through complex emotional situations when needed. Arts therapists who specialize in both arts and mental health are particularly suitable for this role. Overall, the interviewees reported that participation in the integrated arts-based groups positively impacted their personal recovery processes by providing a corrective experience of equality as well as enhancing a sense of belonging to the community and social relationships. The participants also reported being empowered by the final artistic event that not only enhanced their sense of visibility, competence, and aspirations for future development in personal, interpersonal, and artistic realms, but also helped to combat both self- and public stigma.

## Introduction

Stigma and discrimination are the two main challenges faced by people with mental health conditions (MHCs) (United Nations, 2017), and constitute their most significant barriers to recovery (Oexle et al., 2018; Wood and Alsawy, 2018). Public stigma is characterized by negative attitudes toward people with MHCs, including stereotypes, prejudices and discrimination (Corrigan, 2000), and may lead to low willingness to engage in social, occupational, familial, or intimate relationships with people with MHCs (Corrigan et al., 2003). The internalization of such negative attitudes by people with MHCs is termed *self-stigma* or *internalized stigma* (Corrigan et al., 2016), which is associated with a decline in hope, self-esteem, social functioning, self-efficacy (Mittal et al., 2012; Boyd et al., 2014; Yanos et al., 2015; Jahn et al., 2020), and less recovery motivation (Drapalski et al., 2013; Link et al., 2015). The recovery-oriented approach reflects the transition from a medical model of isolation to a social model of integration (Jacob, 2015), places the person at the center rather than the illness, perceives people with MHCs holistically and focuses on their integration in the community by improving their quality of life, sense of control, independence, meaning and hope (Slade et al., 2012; Davidson, 2016). Interventions aimed at reducing self-stigma may increase one’s sense of meaning and promote personal recovery (Ehrlich-Ben Or et al., 2013; Oexle et al., 2018). Consistent with this view, increasing numbers of rehabilitations programs have been designed to integrate people with MHCs into the community. There is evidence that a contact strategy that advocates direct intergroup interaction between diverse populations for changing attitudes (Allport, 1954) is the most effective strategy for reducing mental health public stigma (Corrigan et al., 2012; Ahuja et al., 2017).

The *Amitim* program in Israel enables adults with MHCs to participate in leisure and arts activities that are offered to the general public in community centers across the country (Halperin and Boz-Mizrahi, 2009). *Amitim*’s integrated arts-based groups is a unique model designed to create a meaningful, interpersonal connection between people with MHCs and non-clinical community members who engage in a joint creative activity and take part in discourse about mental health issues. The purpose of this qualitative study was to examine how both participants (people with MHCs and non-clinical community members) experience these arts-based groups and to pinpoint the challenges and benefits.

### The Recovery Approach

“Recovery is a process, a way of life, an attitude, and a way of approaching the day’s challenges. It is not a perfectly linear process” (Deegan, 1988, p. 15). More precisely, *personal recovery* refers to an individual process that can primarily be assessed subjectively by the individual, and is not necessarily related to symptom reduction (Slade, 2010; Chan et al., 2018). In this sense, the process of personal recovery focuses on the individual’s journey rather than on reaching a goal or an outcome. Personal recovery is subjective and unique to each person, and can be an experience of self-discovery, change and personal growth. It requires commitment and optimism from those who have MHCs and their families, as well as a support system from professionals, health services and the community (Jacob, 2015). “CHIME” is a widely accepted conceptual framework to identify personal recovery processes, which consists of five essential components: connectedness (relationships, peer support, and part of society), hope and optimism (positive thinking, motivation, and aspirations), identity (positive self-esteem), meaning in life (meaningful life, goals, and spirituality), and empowerment (responsibility and strength) (Leamy et al., 2011; van Weeghel et al., 2019).

In Israel, as is the case in other countries (Slade et al., 2012; Solli et al., 2013; Fernando, 2014), the recovery-oriented approach has influenced psychiatric rehabilitation policy by shifting care from hospital to community services, as manifested in the 2000 “Rehabilitation in the Community of Persons with Mental Disabilities Law.” This law is aimed at the community rehabilitation and integration of adults with at least a 40% “mental health disability” (as determined by the National Insurance Institute) and enables them to achieve more functional independence and a better quality of life, while maintaining their dignity, in the spirit of the Israeli “Basic Law on Human Dignity and Liberty.” Indeed, this transition has contributed to the quality of life and community integration of people with MHCs (Dudai and Hadas-Lidor, 2008; Roe et al., 2012) and to the establishment of community rehabilitation programs designed not only to reduce self-stigma among people with MHCs, but also non-clinical community members’ public stigma (Roe et al., 2016).

### Community Rehabilitation and Arts-Based Groups

Studies have shown that leisure activities make a positive contribution to the personal recovery process by endowing individuals with a sense of community membership, autonomy, capability, meaning and hope, while reducing depression, stress and boredom (Chan and Mak, 2014; Iwasaki et al., 2015; Fenton et al., 2017; Chan et al., 2018). A recent systematic review indicated that participation in social activities is one of the most common enhancers of social inclusion (Filia et al., 2018).

There is growing evidence that a personal recovery process can be fostered by pursuing activities in various fields of the arts, and that these activities can improve social abilities, connect communities, and promote well-being (Crawford et al., 2013). Lomas (2016) discussed the significant potential of the arts as a means of experiencing growth and fulfillment, and proposed the term of “positive art” to designate the field that encompasses theory and research on the positive effects of arts activities on well-being. His literature review shows that artistic expression in or appreciation of visual arts, music, literature, and drama contributed to five positive outcomes: awareness, enrichment, aesthetic appreciation, entertainment, and connection with others (Lomas, 2016). Leisure activities such as theater or music, when moderated by a professional with an artistic background, enable the participants to perceive themselves as musicians or actors and not as patients (Ørjasæter and Ness, 2016).

Another literature review identified the core components of theater that may contribute to positive change in people with MHCs. These include “processes of group cohesion and affiliation, common goals, common experiences, setting characteristics of openness and inclusion, opportunities for community connections and integration, flexibility, and ownership” (Faigin and Stein, 2010, p. 307). Theater also enables people with MHCs to take on valued social roles and publicly challenge mental health stereotypes (Faigin and Stein, 2010). A qualitative study explored the experiences of people with MHCs, their relatives, and health professionals in a local community theater project where all the participants presented as equal members without explicitly revealing their background (Bosco et al., 2014). The participants with MHCs reported improvement in social relationships and quality of life. The relatives of those with MHCs reported noticing personal growth, empowerment, and reduction in their family members’ self-labeling (Bosco et al., 2014). A different qualitative study of a theater ensemble involving people with MHCs identified several ways in which this community-based theater impacted people with MHCs, including “personal growth through acting, performing as escape from the self, sharing personal stories on stage and the power of personal stories to connect to audiences” (Faigin and Stein, 2015, p. 153).

A narrative study examined the associations between recovery and participation in a theater group that was part of a community rehabilitation program for people with MHCs (Torrissen and Stickley, 2017). Participants in this study reported an improvement in sense of meaning, sense of belonging, social interaction, peer support, and self-esteem. The authors suggested that participation in community-based theater may have a positive impact on the participants as regards all five recovery processes in the aforementioned CHIME model (Leamy et al., 2011). The qualitative findings of a mixed method study reported trends where people with MHCs who participated in playback theater reported improved self-esteem, creativity and self-expression, self-knowledge, as well as experiences of fun and relaxation (Moran and Alon, 2011). The authors argued that consistent with the recovery approach, playback theater encourages people with MHCs to meet as equals with others, promotes contact, facilitates a sense of community, and helps reduce feelings of isolation and stigma. They claimed that “re-authoring one’s own story is the way to shed the identity of “patient” and adopt a new identity with multiple roles” (Moran and Alon, 2011, p. 321). A different playback theater group specially trained to raise awareness of mental health issues gave the audience an opportunity to share experiences of prejudice and discrimination, to protest negative attitudes, and look beyond mental health labels (Yotis et al., 2017). Overall, these studies underscore the contribution of theater to the recovery process. Theater’s rehabilitative power lies in its inherent reliance, as an art form, on the presence of an observing other; theater is often defined as A performs B for C, and “theater” in Greek (*théâtron*) means a “place to behold” “to view” (Theater, 2019).

Music has also been found to impact the recovery process among people with MHCs. According to Ansdell (2010), musical performance not only creates social bonds between people and communities, but also helps to “perform the self” which validates one’s existence; namely, “I perform, therefore I am” (Aldridge, 1996 as cited in Ansdell, 2010, p. 171). A qualitative study examined the effect of a drumming group on people with MHCs, compared to a non-music control group. The findings indicated a decrease in depression, a significant improvement in social resilience, and a significant reduction in anxiety compared to the control group (Fancourt et al., 2016). Another qualitative study examined the contribution of a community-based singing activity to the recovery process of people with MHCs (Lagacé et al., 2016). Participants noted a re-discovery of their identity, increased self-confidence, resuming and engaging in meaningful occupations and projects, improvement in social and cognitive skills as well as in their physical condition. These benefits were attributed to a normalizing environment, lack of stigma, high expectations and support for participants, stress relief activities, emotional expression and arousal of cognitive functions (Lagacé et al., 2016). A qualitative study that included 20 interviews and 2 focus groups indicated that engagement in the community-based singing workshops in “Sing Your Heart Out” (SYHO) project improved or maintained the mental health and well-being of the participants, people with MHCs, as well as the general public (Shakespeare and Whieldon, 2018). A thematic analysis of semi-structured interviews of 25 choir members and 23 creative writing group members with mental health conditions indicated that participating in arts-based groups can be empowering, because it increases the participants’ awareness of their psychological needs such as belonging, support, self-efficacy, purpose and positive emotions (Williams et al., 2020).

With regards to creative writing, it was suggested that “creative writing might play an important role in recovery from mental illness” (King et al., 2013, p. 450). These authors argued that creative writing in the psychosocial rehabilitation for people with MHCs can contribute to personal identity development, the repair of symbolic functions, and the remediation of cognitive functions. They emphasized the importance of the facilitator’s identity as a professional writer rather than a mental health professional in enabling participants to identify themselves as writers rather than as patients (King et al., 2013). The benefits of creative writing in the recovery of people with MHCs were also examined in *Writing for Recovery* (Taylor et al., 2014), a narrative practice development project which provided the participants with a protected space to explore their identities through the writing processes, express their individual unique voices, share social experiences and find personal and social meaning in recovery. In the process of re-storying identities, new relationships were created and the participants’ social and self-confidence increased (Taylor et al., 2014). This is consistent with the view that the process of re-authoring one’s life story is an integral ingredient of the recovery process because it gives the individual an opportunity to regain ownership of his or her personal story (Roe and Davidson, 2005). A phenomenological qualitative study indicated that personal writing (i.e., poetry, creative writing, story-making, or journal writing) may improve the development of self-identity, expand points of view, enhance self-awareness, assist in the understanding of others, enable spiritual transcendence, and promote personal growth and recovery (Haertl and Ero-Phillips, 2019). In sum, the reviewed studies above focused on the potential impact of theater, playback theater, music, singing, and creative writing on people with MHCs- art forms that are offered in the *Amitim* program.

### Integrated Arts-Based Groups in the *Amitim* Program

The *Amitim* program, founded in 2001 by the Israel Ministry of Health and the Israel Association of Community Centers, operates nationwide in about 80 community centers and serves approximately 3,500 adults (aged 18+) with at least a 40% mental disability, who are eligible for rehabilitation services by law. The professional coordinators of the *Amitim* program accompany the adults with MHCs, facilitate their integration in community center activities and support their personal recovery process and psychosocial rehabilitation (Halperin and Boz-Mizrahi, 2009).

*Amitim*’s flagship activities are its integrated arts-based groups which are designed to enable interpersonal contact and meaningful dialogue between people with MHCs and non-clinical community members. The process is focused on a joint creative activity and is accompanied by a structured discussion on mental health issues and the shared experience. Thus, the integrated arts-based groups aim to impart knowledge and skills in theater, music, writing, or other arts, and to facilitate direct interaction between participants. Intergroup contact (i.e., contact between members of different social groups) have been found to be an effective way to reduce prejudice and intergroup tensions as well as to improve intergroup relations (Christ and Kauff, 2019). This format is consistent with Allport’s intergroup contact hypothesis (Allport, 1954), which stipulates that four key conditions are required to create the positive effects of intergroup contact: equal group status within the situation, common goals, cooperation between groups, and support by social and institutional authorities.

*Amitims*’ integrated arts-based groups aim to change attitudes toward mental health in non-clinical community members and to promote personal recovery among people with MHCs. This goal is consistent with the aforementioned cumulative evidence that participating in arts-based groups can have positive psychosocial outcomes. Approximately 15 integrated arts-based groups operate annually in community centers and in several academic settings throughout the country. Each group consists of about 10–25 participants (of whom about 50% are with MHCs), and is led by a group facilitator who is an artist, arts therapist, or other human service professional. Each group is also accompanied by an *Amitim* coordinator. The groups meet weekly for about 2 h and overall group duration ranges between 13 and 36 weeks. A detailed description of the characteristics of participants enrolled in *Amitim*’s arts-based groups is reported elsewhere (Nitzan and Orkibi, 2020).

### The Present Study

This qualitative study is a part of a larger mixed methods research program which includes quantitative data collected at beginning and end of the *Amitim* program’s annual activities. The present report focuses on the qualitative data collected between August and September 2019 from five focus groups to better understand the participants’ experiences in the integrated arts-based groups, as well as the contribution of the groups to the integration of people with MHCs in the community and the reduction of public stigma and self-stigma.

## Materials and Methods

This qualitative study followed two worldviews. The first is a constructive worldview that focuses on participants’ perspectives and the construction of subjective meanings created through interactions with others and cultural norms, as well as the researcher’s interpretation. The second worldview is a pragmatic one that involves multiplicity of methods and different ways of collecting and analyzing data according to the requirement of the research (Creswell, 2014).

### Participants and Procedure

Thirty-eight individuals were recruited from 15 integrated arts-based groups to take part in five focus groups (singing, music, writing, theater, and playback theater). Of the participants, 26 were females (*M* = 42.3, *SD* = 16.48) and 12 were males (*M* = 34, *SD* = 12.83). There were 17 participants with MHCs (females = 12) and 21 non-clinical community members (female = 14). See Supplementary Table 1 for a summary of participants’ demographics.

In May 2019, *Amitim* program research supervisors together with University of Haifa researchers sent a joint letter to the *Amitim* integrated groups coordinators reiterating the importance of focus groups to understand the participants’ experiences and get feedback on the groups. The coordinators were asked to help recruit participants for the focus groups. *Amitim* coordinators of five integrated groups were able to recruit participants for the focus groups and mediated between the researchers and the participants to schedule the time for the sessions. The study was approved by Mental Health Division of the Ministry of Health and by the Ethics Committee at the University of Haifa (approval # 287/17). Participation in this study was voluntary and all the participants signed an informed consent form indicating their willingness to take part in the focus group.

### Data Collection

Focus groups were chosen as data collection method because they constitute a collaborative space which enables an open dialogue between participants following a shared experience. All participants can express their opinions, share personal and collective experiences, exchange diverse perspectives, and offer different interpretations. In addition, focus groups may encourage participants who refrain from one-on-one interview to take part in group interview (Bloor, 2001). Five focus groups were held at the end of the integrated arts-based groups annual activities. The focus groups were documented on sight by a professional typist who was committed to confidentiality. The focus groups adhered to a semi-structured interview protocol with open-ended questions and lasted 90–120 min each. At the end of the focus group, each participant received a gift card as an expression of thanks. See Supplementary Table 2 for the focus group interview protocol.

### Data Analysis

Data were coded using reflexive thematic analysis, which is characterized by theoretical flexibility and is suitable for questions related to participants’ experiences, attitudes, and perceptions (Braun and Clarke, 2020). The process included six phases: familiarization with the data by reading the data repeatedly to be intimately involved in the corpus; coding by identifying key characteristics in the dataset and generating codes; generating initial themes by identifying broader meaning patterns (potential themes) across codes in the collected data; reviewing themes by examining candidate themes checked against the dataset and refining them if needed; defining and naming themes by analyzing each theme in detail and giving an informative name to each theme; writing up the report by weaving the data extracts together with the narrative. The checklist for conducting a good thematic analysis was used as a guide to ensure an analysis that was rigorous and robust (Terry et al., 2017; Braun and Clarke, 2020).

## Findings

The findings consist of three main themes that are shown in Table 1.

**TABLE 1:** The resulting themes.

**Theme 1.** Creation and expression through the arts promote well-being	**Theme 2.** Self-disclosure in a safe space encourages sense of belonging	**Theme 3.** We are all in the same boat
1.1. Release and emotional expression	2.1. A safe space enables self-disclosure	3.1. The group as a corrective experience
1.2. Introspection and self-discovery	2.2. Self-disclosure encourages belonging	3.2. Equality: we are all “copers”
1.3. “Just like therapy”		3.3. The final performance as a shared experience of visibility and competence
1.4. Experience of spiritual elevation		
1.5. Interpersonal communication		

### Theme 1: Creation and Expression Through the Arts Promote Well-Being

#### Release and Emotional Expression

Many participants indicated that creation and expression through the arts generate a sense of freedom, release inhibitions, and allow for the expression of emotions that would not have been expressed in other ways. Kevin (pseudonym), for example, referred to the ability of the arts to fully express personality: “I think each art form releases and brings out someone’s personality, his endless ability, it releases him from everything” (28/Singing/MHCs)^1^. Carl described the way in which music helps to overcome negative experiences: “It [music] releases many things, mainly negative, that have been accumulated… and there are certain songs you identify with and feel as if they were written about you…” (23/Music/CM). Lily referred to the ability of writing to touch emotions: “The beauty of writing is the emotion that comes out without barriers… it just comes out, everyone brings out their own pain, their loss, the things that make them feel, whatever makes them cry” (37/Music/MHCs).

#### Introspection and Self-Discovery

Many participants indicated that creation and expression through the arts promotes introspection and enables self-discovery. Sheila, for instance, said: “Music allows me listen to myself, to my desires, to my needs, I know what is right for me” (48/Singing/MHCs). Claire spoke of unconscious elements and new insights that emerged from writing: “All of my writing… I brought out my other self, what was hidden… My writing focused on the duality within me” (46/Writing/MHCs). Talia described her self-discovery process: “You tell the story with words, and very slowly you bring out your own personality… and you were unaware it existed within you… at first these are simple words but afterward you find your secret…” (66/Playback/CM). Carl shared his insights as a result of the creative process: “I wrote a letter to my dad, about taking responsibility for mistakes I made and paying the necessary price for them… and this is something that was very meaningful in my life…” (23/Music/CM). His groupmate, David, added excitedly: “I wrote a letter to my deceased uncle and I discovered that because he loved the sea, it is hard for me to go to the sea… I just cried and went to the sea after more than 5 years of avoiding it” (26/Music/MHCs).

#### “Just Like Therapy”

Many of the participants stated that creation and expression through the arts feels “just like therapy.” Vera referred to music as creating an internal healing process: “You relish in something through your sense of hearing and it heals you from within, activates emotions, processes them and connects to them… it begins a process of healing…” (39/Music/CM). Sheila described the act of singing as relaxation: “You start singing and it’s as if your body relaxes, it’s a kind of therapy” (48/Singing/MHCs). Nancy referred to the writing process as a gateway to the inner world: “I think there’s something therapeutic in putting forward something that is so personal… words allow access to my inner world” (55/writing/CM). In contrast, her groupmate, Anna, pointed to the danger of overexposure: “When a group becomes something more therapeutic it loses its value… because therapies, with all due respect, we have had enough…” (38/writing/MHCs).

#### Experience of Spiritual Elevation

Many of the participants referred to the spiritual aspect of creation and expression through the arts, which is beyond the spoken word or rational thinking. It is an elevating experience of connecting to the powerful and divine spring of creativity. Kate tried to explain the spiritual experience music affords: “Music takes us to another dimension, to a kind of spiritual level that we cannot achieve during a conversation, because it’s hidden very deep within us and we cannot express it in words… with music, suddenly something comes into the surface” (25/Music/CM). Peter found it difficult to describe the unique experience in words: “Everyone knows what it’s like to feel something, and no matter how many words you use to try to describe it, it won’t convey the feeling… music… it’s more a connection on the divine level” (26/Music/CM). Ellison described an unexpected mystical process: “I see it as a mystical consciousness process… I don’t know what I’m going to be and what will pass through me… it’s surprising every time” (41/Playback/CM), and Kevin emphasized the unique experience of elevation, which is hard to explain in words: “The moment you create something… you’re above your own self… there’s power in a choir, there’s power in singing, I think it’s something that is very hard to explain, in the same way that you cannot explain in words what love is, it’s something you feel” (28/Singing/MHCs).

#### Interpersonal Communication

Many participants referred to creation and expression through the arts as a unique channel of communication which helps them communicate with their environment, encourages social encounters, and alleviates loneliness. Vera, for example, described the social changes she experienced because of the process: “The words, the music, the sounds carry you… I was a person who afraid to make her voice heard or express herself… so not only have I overcome shyness, but I also feel socially accepted” (39/Music/CM). Maria described the unmediated way in which theater allows expression: “The theater allowed me to speak and bring out everything I feel in a different way… once you manage to speak… people also see you differently” (33/Theater/MHCs). Fred referred to the way in which creation and expression through the arts mediate the inner and the outer worlds and make personal content more accessible: “You learn to deliver this content, what you produced … so, it is both readable, interesting, something that someone would like to read” (57/Writing/CM). Similarly, Nancy referred to writing as enabling communication with others: “Writing, at its core, is a communication of repressed issues… I wrote something that led me to understand something incredibly significant about myself… before reading, I said to myself: “It is entirely uncommunicative”… but… wow… everyone understood me” (55/Writing/CM).

### Theme 2: Self-Disclosure in a Safe Space Encourages a Sense of Belonging

#### A Safe Space Enables Self-Disclosure

Most of the participants referred to the group as a safe space that provided a sense of unconditional acceptance that permitted them to bring up complexities without fear of judgment or criticism. Selina explained the need for a safe place: “There must be a safe space for us, to be exposed and bring out our psyche materials… to come every time and bring part of yourself, knowing it will be embraced and accepted in this room” (60/Writing/CM). Ella mentioned the importance of containment for musical development: “I wanted a place that can contain and hold [me]… to progress with music in a safe environment and I got more than I expected…” (21/Music/MHCs).

According to many participants, the feeling of safety was influenced not only by the participants themselves, but also by the facilitator’s presence and his/her ability to contain the group while maintaining clear boundaries. Ellison, for instance, emphasized the facilitator’s presence: “She was very present and when you feel her presence you can feel safe around her…” (41/Playback/CM), and Natalia clarified the importance of setting boundaries: “Wherever a strong hand was needed, she took a strong position” (53/Playback/MHCs). James described the facilitator’s ability to hold the group in a positive way: “She knew how to set boundaries, but with a smile” (46/Singing/MHCs). Bill commended the facilitator’s ability to deal with members leaving during the process: “The way in which the facilitator dealt with it, was very concrete, very compassionate, but also very respectful, and it was clear to all of us that whoever was left, was in good hands” (25/Theater/CM). In contrast, Mali, from the same theater group, who was negatively affected when participants left the group, suggested that help from an outside professional was necessary: “I found it very hard when people left, and I felt that there wasn’t enough of a discussion about it… someone from outside the group could have come and talked about it… I didn’t know how to deal with it” (26/Theater/CM).

Some non-clinical community members in the music group expressed the need to be accompanied by a professional to contain complex mental experiences. For example, Peter emphasized the need for more preparation in terms of what they would encounter, to be able to support their groupmates when needed: “Here it is critical… to know more about people. So, if someone says, “I have a manic-depression condition,” or someone else would say, “I have an outbreak”… I would be ready for it. I want to be his friend and I’m ready to contain it” (26/Music/CM). Conversely, participants in the writing group thought the facilitator avoided over-disclosure and dwelling upon “deep psychological issues” because the facilitator focused on the artistic form rather than the content. Selina stated that “The facilitator held the safe space and indeed… she did not focus on the content of the psyche depths of each participant rather [just] the essence of it all. The constructive criticism referred to the form” (60/Writing/CM). Diana addressed the dangers inherent to over self-exposure: “One needs to avoid falling into holes” (72/Writing/CM), and Fred described the clear boundaries set by the facilitator: “[the facilitator] made sure the group did not go in the wrong direction…. if someone started to analyze the content, she put a stop to it” (57 Writing/CM). Nancy concluded: “I think she [the facilitator] succeeded, without making it a therapeutic group, in creating a space in which different contents were brought to the surface… a space which is very open minded, a space that allows you to explore everything for real, not in a sense of “solve my problem,” but simply “I wrote about it, and I’m sharing it with you…” (55/Writing/CM)”.

#### Self-Disclosure Encourages Belonging

Self-disclosure encourages a sense of belonging and acts as a “ticket” to the group. Lily shared her insights: “Openness creates openness, period. The question is how to achieve this initial openness…. You cannot force people to say things” (37/Music/MHCs). Faith emphasized the importance of sharing in the group, as a way of instilling a sense of belonging: “I had the opportunity to tell my life story since the day I was born… and it enabled me to feel comfortable with everyone… Once you reveal your past, your history, you become a part of the group” (67/Singing/CM). Dan described the process of creating a sense of belonging in the group: “I, for example, do not belong to *Amitim*, however, I also have my difficulties… at first it was hard for me to talk about it, but I saw that it’s common to so many of the group, so I felt a part of it… that I can also share and open up… I felt brave and strong afterward because I could do it in front of everyone” (29/Singing/CM).

Many participants described the empathy they felt toward their groupmates because of their disclosure. This feeling deepened their mutual sensitivity and created a fertile terrain for commitment to the group and the process. Sheila described the impact her story had on the other members: “My story probably touched [the participants] and it allowed people to speak up” (48/Singing/MHCs). Carl stated how he identified and empathized with others in his group: “I feel I had someone to identify with… as if I can share this empathy with more people (23/Music/CM), and George talked about the experience of being together: “You tell your story, and the whole group tries to help you, and look for ways to reach out, or how to touch, or where it touched them…” (59/Playback/CM).

### Theme 3: We Are All in the Same Boat

The group was compared to a boat sailing on the water with everyone on board. The weather is constantly changing, the sea is sometimes calm and at other times stormy, strong winds are blowing and currents of water from different directions threaten to capsize the boat. The boat reaches the shore, thus culminating the journey in performance. During this emotionally challenging journey, the group members learn to listen to themselves and to others, to take space as well as to give, and utilize the artistic medium to look within and outside into the world, look straight in the eyes of their community.

#### The Group as a Corrective Experience

Many participants said they went through a new experience which differed from past group experiences. Ella said “Honestly, [it] is the best experience I’ve been through in my whole life… For me, it corrected what I went through at school: the attitude, my feelings in the group. I always had anxieties in groups that I would re-experience the same… After I said this a few people hugged me, and I saw that it’s different here and I feel accepted and loved and can express myself…” (21/Music/MHCs). James described a new experience of connection and support: “I truly discovered genuine support for one another, and a bond which didn’t exist in any other group I’ve ever been to, because there were always ego and power struggles” (46/Singing/MHCs). Dan likened his experience as a youngster in an adult band, to his current experience: “They didn’t give me opportunities to sing solo… they put me in the back … it crippled my motivation to continue. When I came here, I felt there is no discrimination against someone older, advanced, beginner… I’m given my own place and the atmosphere is a lot more supportive…” (29/Singing/CM). Sofia described the changes she experienced: “Usually, when I join a group, I’m very reserved and quite scared of what others will say about me. But here, when I saw how they approached me in the first session, I said to myself: “Go ahead, raise your hand, don’t be shy”… I came home and told my mom: ‘Why can’t every day be a Thursday?”’ (22/Theater/MHCs). Jack described his experience as entirely different from anything he had experienced so far in similar situations: “When I went on stage I did so with tears in my eyes…That for me was the most significant moment…Usually, when I’m feeling unstable, I move away from people, whereas over there [on stage] I put myself in front of people” (33/Theater/CM).

#### Equality: We Are All “Copers”

Most of the participants with MHCs felt they did not experience prejudice when getting acquainted with their groupmates, but rather were accepted “at face value.” They emphasized the importance of getting to know one another without stigma. They also indicated they are often guided by a sense of mission to normalize difficulty and show the non-clinical community members that they are not defined by their illness. Some of the participants with MHCs admitted that there is something comforting in discovering that non-clinical community members also need to cope with mental health issues. Kevin stated: “Even those who are not diagnosed are coping with issues, even those who do not seem like people with MHCs suddenly tell us about things they’ve been through, difficulties they have, and it is very much encouraging for us… I am here as a messenger; to show we are part of the community… And overall, we are completely ordinary people, the illness does not define us” (28/Singing/MHCs). Sheila confirmed this by stating: “We are humans first [and foremost]. We are not our illness” (48/Singing/MHCs). Anna stated: “It’s not like we have a sticker on our forehead: “I’m coping with a mental condition,” “I’m ordinary”…. You wouldn’t know who’s coping with a mental issue and who isn’t” (38/Writing/MHCs). Nina emphasized her self-perception as an ordinary person: “I came to tell my story, showing that even though I’m coping with something, I don’t have horns, or a tail, or anything, I’m an ordinary human being. Just like there is someone coping with a physical disability, I am coping with a disability which is a mental one… and indeed I was worried… that there would be “them” and “us” – “the copers” – but it was not the case” (28/Theater/MHCs), and her friend Maria concluded: “I think that what allowed us to love one another is that we learned to get to know each other without the stigma…” (33/Theater/MHCs).

Similarly, many of the non-clinical community members maintained they did not always know who had an MHC and who did not, everyone has vulnerabilities and complexities, and labeling is irrelevant, as reflected in Ellison’s words: “I never understood or knew which participants had mental illness and who didn’t. We were all “copers” (41/Playback/CM)”. Some of the non-clinical community members described a process that reflected a change in perceptions from fear of problems with opposing groupmates to a realization that “we are all copers” and equals. Isabella clarified: “At the beginning I felt certain disparities but with time it turned into a “non-issue”. I didn’t care who’s from *Amitim* and who isn’t … you hear someone’s story, and you don’t know how to deal with it… which is often a strange world for me. So, there was a certain shock and difficulty… but with time… it passed” (45/Playback/CM). Bill, who had just begun taking psychiatric medication stated: “I realized that I have an mental health condition too, so it’s really frightening. I didn’t want to see people who are like me, I was very afraid it would make me deteriorate… and this year made me feel the best I ever felt, this group…” (25/Theater/CM).

Different points of view expressed by the participants with MHCs and non-clinical community members brought the issue of “us” and “them” to the surface. For example, Emily divided the participants into those who are healthy and those who are sick, those who are strong and those who are weak, those who help and those who need help and was convinced that the “other participants” were told to help those who experienced difficulties: “I felt tremendous support from them… They helped and supported me throughout which was amazing, and I believed that they had been instructed to ‘go help this one”’ (56/Theater/MHCs). Faith apparently differentiated between herself and the participants with MHCs stating “the encounter gave *them* a lot” (67/Singing/CM).

The sense of equality also stemmed from the fact that the facilitators provided equal opportunities for all participants regardless of their mental health issues, Nancy stated: “There is something about her [facilitator] that made everyone [feel] equal… she said more than once that she is also a “coper” like the rest of us…. everyone starts at the same place, regardless of background and life circumstances and this and that category”… (55/Writing/CM). Claire confirmed: “She treated everyone equally rather than who’s a better writer… she truly gave everyone the stage and a warm, encouraging attitude…” (46/Writing/MHCs). The group facilitators set high artistic standards for all participants and in this way conveyed a message of equality that everyone can face challenges. George stated: “she demanded high standards. Like, if something was not accurate… then she motivated us or insisted on it. She always made us feel like we needed to appreciate ourselves as actors” (59/Playback/CM). His groupmate, Liliana, added: “She [the facilitator] believed in each one of us… we came with a certain goal we all shared … I felt real warmth from everyone who were united around a certain goal … and there was equality among all of us” (26/Playback/MHCs).

#### The Final Performance: A Shared Experience of Visibility and Competence in Front of the Community

The culmination of the group process was a performance in which the participants share the output their creation with the community, family, and friends. The participants were committed to the final performance which made them feel visible and seen. Many of the participants with MHCs described being in front of the audience as a coming out, moving from the darkness into the light. David stated: “It’s like coming out of the closet… as if until now we were hidden inside us… in a performance you reveal yourself in front of everyone” (26/Music/MHCs). Kevin stated: “You are literally being seen, both as a participant with MHCs, as well as a vocalist and it’s fun. They [the audience] come… and they suddenly see: “Wow… you are regular people”… I said [to myself]: ‘I broke the stigma, I won!”’ (28/Singing/MHCs).

Many participants with MHCs argued that the belief in their artistic ability allowed them to do things and produce pieces they did not believe they could produce. They emphasized the experience of success and the feeling of competence they felt after the performance. Emily admitted: “I simply didn’t think I’d be capable of doing it… they made me feel I was worth something after all… it gave me meaning… I felt love…” (56/Theater/MHCs). Ella said she “felt it was the climax of the whole process …” (21/Music/MHCs).

However, two participants with MHCs from the theater group were dissatisfied with the drastic transition from the more relaxed work process that characterized the meetings during the year, to the pressure and intensiveness that characterized the preparations for the final performance. Emily stated: “All of a sudden there was an insane pressure for the performance, suddenly we come on Fridays-Saturdays [weekend], the whole week, every day, and it was like completely unbalanced… it was very… for me… intensive… It was extremely hard” (56/Theater/MHCs). Maria agreed, and added: “It became stressful… also because I’m working and if I don’t work, I won’t have money for rent or for therapy” (33/Theater/MHCs).

The non-clinical community members discussed the positive experience. Kate addressed the empowering experience of being seen: “Suddenly we are on stage and there are people from the outside who see us, and all of a sudden from being in a room, we are in a whole auditorium, [they] see what we were doing the whole year…” (25/Music/CM). Jessica stated: “There was something crazy in the air… I felt as if the auditorium was very small suddenly… it was such an intense experience, and I was very excited… there was truly a strong bond” (26/Theater/CM). However, Diana expressed her concerns: “I invited my son to the performance… I was very afraid of the criticism… but he was extremely impressed… it was very dignified [performance]…” (72/Writing/CM).

## Discussion

The purpose of this study was to document the experiences of participants in *Amitim*’s integrated arts-based groups and to pinpoint the challenges and benefits. The findings suggest that *Amitim*’s integrated arts-based groups fulfill the four main conditions to create positive effects of intergroup contact (Allport, 1954): participants with MHCs and non-clinical community members met as equals, there was intergroup cooperation and work toward a common goal, with the support of the *Amitim* program and the National Insurance Institute (i.e., the “authorities”). The participants with MHCs reported they did not experience any manifestations of prejudice, consistent with findings showing that contact is the most effective strategy for reducing public stigma in adults (Corrigan et al., 2012; Ahuja et al., 2017).

For the participants with MHCs, the integrated arts-based groups were an opportunity to normalize their difficulties. Specifically, the groups fostered the feeling that their illness does not define them and enabled them to experience more intergroup similarities than differences, similar to other studies where people with MHCs interacted with others as equals, which helped reduce feelings of isolation and stigma (Moran and Alon, 2011). In some groups, the non-clinical community members stated they had no idea which participants had MHCs and which were non-clinical community members, while in other groups some participants indicated that over time it became a “non-issue.”’ In one group, a non-clinical community member who started to take antidepressants wondered if he should now belong to the group of participants with MHCs, and some participants with MHCs were surprised to discover that non-clinical community members also cope with mental health issues. This finding underscores the blurred intergroup differentiations in *Amitim*’s integrated arts-based groups that meaningfully contributed to the overall sense of equality and acceptance. This suggests it is crucial to facilitate acquaintance between participants as equals, without labeling or prejudice, thus permitting each individual to decide when and if to reveal their condition. However, in our study, few participants with MHCs in the integrated groups still expressed internalized negative attitudes (self-stigma), which seems to have impacted the way they have experienced their encounter with the non-clinical community members.

### The Role of the Facilitator

Most participants indicated that the facilitators provided equal opportunities for all of them, regardless of their mental health status. The facilitators made equivalent demands of high artistic quality and commitment to the artistic process, which contributed to the sense of equality experienced by the participants with MHCs.

The facilitator’s identity was perceived as a significant factor for the participants. The music group was facilitated by a well-known musician, the singing group, by a singer, the writing group by a writer, the theater group by both a theater director and psychodrama therapist, and the playback group was facilitated by a drama therapist. On one hand there are many advantages to having facilitators who are well-known professional artists in that they have the skills to support the creative process and set the same standards for everyone. Studies have shown that the facilitator’s identity as a professional with an artistic identity, rather than a mental health professional, allows participants to identify as writers, actors, or musicians rather than as patients (King et al., 2013; Ørjasæter and Ness, 2016). On the other hand, an artist is probably less well equipped with the knowledge and skills to deal with situations that may arise when group members experience personal or interpersonal difficulties. The consistent presence of a mental health professional in the group, such as the *Amitim* coordinator, may provide an adequate response. The need for the presence of a mental health professional can be resolved by assigning an arts therapist who has been trained in arts and therapy as a facilitator, as was done in a playback theater group in which some of the actors were also therapists (Yotis et al., 2017). Interestingly, the need for the involvement of a mental health professional was voiced to a greater extent by the non-clinical community members than participants with MHCs; possibly because the latter are more familiar with mental health issues and are undergoing treatment. In fact, the non-clinical community members’ need for emotional support also contributed to the sense of equality in participants with MHCs.

### The Benefits of the Arts

Most participants felt that creation and expression through the arts promote well-being, consistent with findings that engagement in community-based arts groups promote well-being (Shakespeare and Whieldon, 2018), social connectedness and the process of personal recovery (Crawford et al., 2013). Opportunities for community connections and inclusion are recognized as among the core components of theater that may contribute to positive change in people with MHCs (Faigin and Stein, 2010). The participants referred to the creation and expression through the arts as a unique channel of communication which mediates the inner and outer world, allows for self-expression, and focuses on strengths rather than the illness. They emphasized the arts’ unique qualities that enable introspection, self-discovery and insights as the creative process unfolds. Disclosing personal contents in the groups encouraged mutual empathy and promoted a sense of belonging. The participants had the opportunity to share their emotional experiences in a safe space, while experiencing acceptance, containment, and support. This is consistent with the idea that “re-authoring one’s own story is the way to shed the identity of ‘patient’ and adopt a new identity with multiple roles” (Moran and Alon, 2011, p. 321). Similarly, research on community-based singing activity suggests that people with MHCs experience re-discovery of their identity (Lagacé et al., 2016). This is consistent with the recovery-oriented approach which perceives people with MHCs holistically, places the person in the center rather than the illness (Slade et al., 2012; Davidson, 2016), and views the recovery process as a subjective journey of self-discovery, change and personal growth (Jacob, 2015).

Many participants referred to creation and expression through the arts as a mystical process of consciousness, spiritual elevation, and a sublime experience of connection to the powerful divine spring of creativity. Similarly, research indicates that people with MHCs experienced personal writing as enabling spiritual transcendence (Haertl and Ero-Phillips, 2019). Transcendental or “divine” awareness can be expressed in poetry, music, or other arts (Mitchell, 2011), and is associated with hope, empowerment, and a sense of well-being (Tanyi, 2002). Spirituality was noted to be inherent to the rehabilitation process of people with schizophrenia and their well-being (Ho et al., 2016), and is one of the significant factors supporting the recovery journey (Wood and Alsawy, 2018). This is consistent with the CHIME framework for personal recovery processes, where spirituality is part of having a sense of meaning in life (Leamy et al., 2011). Hence, the spiritual experience described by the participants and expressed in creation and expression through the arts may play a central role in the process of personal recovery.

Some participants experienced the creation and expression through the arts to be “just like therapy,” consistent with the findings that participating in arts-based groups can be empowering, because of the participants’ increasing awareness of their psychological needs (Williams et al., 2020). However, a participant with MHC objected that the group could become therapeutic because “there are already enough therapies.” This view emphasizes the need to preserve the group as a place for artistic development accompanied by an in-depth process of growth and personal empowerment. Nevertheless, different participants have different needs and groups differ in terms of their level of disclosure, need for support, and the ability to contain the intensity and pressure associated with the final performance. Still, it would be useful to compare the impact of integrated arts-based groups to that of creative arts therapies (Orkibi et al., 2014; Orkibi and Feniger-Schaal, 2019; Feniger-Schaal and Orkibi, 2020; Shafir et al., 2020).

Some of the participants described the final performance as “a moving from the darkness into the light” and referred to the empowering experience of “being seen” or the “performance of the self” which validates one’s existence (Ansdell, 2010). The sense of being transparent causes people with MHCs to hide from society (Corrigan et al., 2016; Jahn et al., 2020), thus the experience of “being seen and being heard” was a particularly empowering experience for the participants with MHCs. The final performance was an opportunity to reveal themselves to their family, friends, and community, to share the healthy parts, their abilities, vitality, and creativity. They are equal human beings just like the non-clinical community members who perform with them. The audience may change their perception focused on illness and inability, and see them in a more positive light, which may help reduce public stigma. According to the participants, experiencing competence and success is a corrective experience that helps to increase self-confidence, self-esteem, and a sense of self-efficacy, and may reduce self-stigma. The path to further successful experiences, even in everyday life, has been charted.

### Limitations and Future Directions

While the study consisted a sizable number of participants, it only included five focus groups, out of 30 integrated arts-based groups, and only included individuals who volunteered to participate in the focus groups, rather than all members. Future studies would benefit from interviewing people with MHCs in *Amitim* who chose *not* to participate in the integrated groups to learn about potential barriers and hindering factors. Furthermore, the only clinical information available from *Amitim* was that the participants had a mental health disability of 40% or more. While this approach is consistent with the non-labeling stance of the recovery model of *Amitim*, as well as the ethics approval obtained for this study, it would be useful to assess how individuals with different MHCs (i.e., diagnoses) experience the program. Future studies should also examine the unique meaning of each artistic medium *per se* and arts forms not included in this study, such as visual art and dance. Future work could examine the associations between spirituality, creation and expression through arts, mental health, and recovery. Finally, our quantitative findings of pre- and post-participation in the integrated arts-based groups would shed light on the influence of the program on stigma reduction, sense of community, and personal recovery.

## Conclusion

The findings suggest that according to the interviewees participating in integrated arts-based groups had a positive impact on their personal recovery processes in a variety of ways including a sense of belonging to the community, motivation, and aspirations for future development in personal, interpersonal, and artistic realms, introspection, increased self-esteem and self-efficacy, and a greater sense of meaning and empowerment. These findings are consistent with the “CHIME” framework that defines components of personal recovery (Leamy et al., 2011). Participants’ experiences in the integrated arts-based groups underscore the role of creation and expression through the arts in emotional expression, spiritual elevation, self-discovery, interpersonal communication, and ultimately, their well-being. Furthermore, in the two groups that were run at educational institutions where the non-clinical community members were students, the integration of participants with MHCs into an educational program had two benefits: (a) instilling confidence in participants with MHCs that they can enroll in educational programs open to all, and (b) helping to combat public stigma, prejudices, and discrimination. Thus, the expansion of integrated arts-based groups to more educational institutions in addition to community centers should be considered for a more inclusive educational curriculum. Facilitators of integrated groups should create a sense of equality by enabling intergroup acquaintances without required or premature disclosure of the participants’ mental health status. Because arts-based group facilitators must be able to contain and work through complex emotional situations when needed, it seems safe to conclude that arts therapists, who specialize in both arts and mental health, are particularly suitable for that role. When an artist facilitates an integrated group, he or she should be accompanied by a mental health professional. Thus overall, these programs help optimize not only the fight against stigma in the community but also support the personal recovery journey of all their members.

## Data Availability Statement

The datasets presented in this article are not readily available because the qualitative data from focus groups are in Hebrew. Requests to access the datasets should be directed to corresponding author.

## Ethics Statement

The studies involving human participants were reviewed and approved by the Human Research Ethics Committee, Faculty of Social Welfare and Health Sciences, University of Haifa (approval # 287/17). The patients/participants provided their written informed consent to participate in this study.

## Author Contributions

AN: study design, data collection and analysis, and writing and revising of the manuscript. HO: study design, writing and revising of the manuscript, and supervision. Both authors contributed to the article and approved the submitted version.

## Conflict of Interest

The authors declare that the research was conducted in the absence of any commercial or financial relationships that could be construed as a potential conflict of interest.

## Footnotes

1 Participants’ details are indicated in parentheses after the quotation: Age/Group/MHCs or CM for “non-clinical community members”.
