# Supplementary material for: “We’re All in the Same Boat” – The Experience of People With Mental Health Conditions and Non-clinical Community Members in Integrated Arts-Based Groups
Source: Front Psychol. 2021 Mar 17;12:661831. doi: 10.3389/fpsyg.2021.661831 (PMC8010183; doi:10.3389/fpsyg.2021.661831)
Supplement: Supplementary file 1 [file Data_Sheet_1.pdf]

Frontiers in Psychology
https://doi.org/10.3389/fpsyg.2021.661831

“We’re All in the Same Boat” – the Experience of People with Mental Health Conditions and Non-Clinical Community Members in Integrated Arts-Based Groups

Aya Nitzan & Hod Orkibi (2021)

Online Supplemental Material

Table S1.

Focus Groups Participant

Participant's number and pseudonym	Age	Gender	Group	Participant status
1. Sheila	48	F	Singing Group	Participant with MHC
2. James	46	M	Singing Group	Participant with MHC
3. Kevin	28	M	Singing Group	Participant with MHC
4. Maria	33	F	Theater Group	Participant with MHC
5. Nina	28	F	Theater Group	Participant with MHC
6. Emily	56	F	Theater Group	Participant with MHC
7. Sofia	22	F	Theater Group	Participant with MHC
8. Ella	21	F	Music Group	Participant with MHC
9. Lily	37	F	Music Group	Participant with MHC
10. David	26	M	Music Group	Participant with MHC
11. John	23	M	Music Group	Participant with MHC
12. Claire	46	F	Writing Group	Participant with MHC
13. Anna	38	F	Writing Group	Participant with MHC
14. Julia	53	F	Writing Group	Participant with MHC
15. Liliana	26	F	Playback Theater Group	Participant with MHC
16. Natalia	53	F	Playback Theater Group	Participant with MHC
17. Adam	33	M	Playback Theater Group	Participant with MHC
18. Faith	67	F	Singing Group	Non-clinical community member

Frontiers in Psychology
https://doi.org/10.3389/fpsyg.2021.661831

**Participant's number and pseudonym**	**Age**	**Gender**	**Group**	**Participant status**
19. Dan	29	M	Singing Group	Non-clinical community member
20. Mali	26	F	Theater Group	Non-clinical community member
21. Jessica	26	F	Theater Group	Non-clinical community member
22. Lora	25	F	Theater Group	Non-clinical community member
23. Jack	33	M	Theater Group	Non-clinical community member
24. Bill	25	M	Theater Group	Non-clinical community member
25. Ruth	22	F	Music Group	Non-clinical community member
26. Vera	39	F	Music Group	Non-clinical community member
27. Kate	25	F	Music Group	Non-clinical community member
28. Carl	23	M	Music Group	Non-clinical community member
29. Peter	26	M	Music Group	Non-clinical community member
30. Diana	72	F	Writing Group	Non-clinical community member
31. Selina	60	F	Writing Group	Non-clinical community member
32. Nancy	55	F	Writing Group	Non-clinical community member
33. Fred	57	M	Writing Group	Non-clinical community member
34. Isabella	45	F	Playback Theater Group	Non-clinical community member
35. Ellison	41	F	Playback Theater Group	Non-clinical community member
36. Camilla	70	F	Playback Theater Group	Non-clinical community member
37. Talia	66	F	Playback Theater Group	Non-clinical community member
38. George	59	M	Playback Theater Group	Non-clinical community member

Note. MHC = mental health condition; NCM = non-clinical community members.

Frontiers in Psychology
https://doi.org/10.3389/fpsyg.2021.661831

Table S2

Focus Group Interview Protocol

1. How did you get into the *Amitim* program? (through personal connection, volunteering).
2. Do you participate in more *Amitim* programs' activities?
3. What led you participating in the integrated group, and why did you choose this art medium? (theater / singing / music/ writing/playback)
4. What do you think is the unique contribution of the arts?
5. What can you say about the discourse on mental health in the group? Was this discourse reflected in the art works, and how?
6. Would you prefer the group participants to know who is from Amitim and who is not, or to remain anonymous, and why?
7. How did you experience the group facilitator?
8. What was the most meaningful experience for you?
9. What could you recommend for improvement or change?
10. Is there anything else you would like to address?
